# Race realism goes both ways

**DOI:** 10.1007/s40656-024-00658-y

**Published:** 2025-02-10

**Authors:** Rob DeSalle, Ian Tattersall

**Affiliations:** American Museum of Natural History, New York, NY 10024 USA

**Keywords:** Race, Racism, Racialism, Philosophy, Domain problem

## Abstract

We examine the philosophy of race from the perspective of the identified problems with such a philosophy – domain problems, deference problems and mismatch problems. Any philosophy of race should consider at least two domains of human endeavor – the social and the natural. In most cases the social domain defers to the natural domain for a biological explanation for race. Some researchers suggest that there is an impasse in the natural domain that keeps the door open for a biological explanation of race. We examine this purported impasse and conclude that there is a complete lack of scientific support for the existence of human races, and that hence the impasse is a mirage. The inference of no biological races is not, however, a non-result. The consequent lack of support for biological races can be acknowledged by social scientists in formulating their social domain-oriented ontology of race.

## Introduction

In recent years, a philosophy of “race” has been developed by researchers intent on clarifying this important but hugely misunderstood term/concept as it applies to human beings. This philosophy (Ludwig, 2021; Zack, 2018) attempts to reconcile findings in the social and natural sciences, the two areas of investigation most commonly involved in elaborating our conceptions of race. If this approach is to be meaningful, three major problems need to be satisfactorily addressed: the domain problem, the deference problem, and the mismatch problem. Without attention to each and all of these, we cannot develop a meaningful ontology of human race.

## Racial realism goes both ways

The first of the three problems, that of domain, concerns the placement of the background knowledge used to develop an understanding of human race into one scholarly domain or another. The relevant domains in this instance are the natural and social sciences. The argument is that, if race requires an ontology based on natural principles, then that ontology needs to be built on biological background information that may in turn be used by social scientists to develop a fuller and more accurate account of what (if anything) human races are. In other words, if (and only if) there is a biological basis for race, then biological knowledge needs both to inform the social ontology of race, and to provide the basis for its further ontological development in the social domain. All of this assumes, of course, that there is indeed a biological or natural basis to race – failing which, biological/natural notions of race clearly should not be used in the overall ontology. Indeed, if there is no biological basis to what we perceive as race in humans, this fact should not be considered as “non-information,” but rather as knowledge that is foundational to any social ontology of race.

The two other problems, deference and mismatch, spring directly out of the domain problem. The “deference,” (or “expertise”) problem involves one domain of investigation (say, the social) deferring to a second domain (say, the natural). This problem arises when, in order to develop an effective ontology, one domain defers to expert resolution in another. In the present case, many social scientists have deferred to biological experts when attempting to understand the underlying nature of race, so that explanations developed in one domain have guided how subsequent philosophical attempts at an ontology have been conducted in a different one. Clearly, allowing such inter-domain deference has its dangers, especially when issues in the adjacent domain are unresolved or disputed. In the case of human race, biological background knowledge and even the semantics of biology have been used as tools by the developers of social ontologies. The mismatch problem, in contrast, occurs in cases where two or more domains contribute to an overall ontology, but the bodies of knowledge (in total or in part) generated in the various domains are not in agreement. The philosophy of race appears to have suffered from both the domain and the deference problems; and, if allowed to, it could also suffer from the mismatch problem (see below).

Philosophers of race seem to have relied on two assumptions about the domain problem. The first is that researchers in the social domain will always defer to the experts in the natural domain when it comes to an ontology of race, and that this deference is a one-way street. The second concerns the presumption by social scientists that research in the natural domain is ambiguous in regard to the existence of race (Andreasen, 2000, 2005, 2020; Kitcher, 2007). There are many problems with each of these postulates when developing a social ontology of race. First, it is not necessarily true that developing social ontologies requires a natural definition of race. This is partly because of the mismatch problem. If the conclusions of a natural ontology of race are at odds with those of a social ontology, then under common assumptions resolution of the mismatch requires deference one way or the other. Nonetheless, it is still true that the social ontology can be developed independently of the natural domain without the exercise of deference; and indeed, the natural existence of race may not necessarily contribute significantly to our apprehension of the social ontology of race. To understand this, we need to examine what a biological or natural perspective on race actually contributes to a social formulation of race, for it may simply be that a natural definition of race is irrelevant or only minimally relevant to the social ontology of race. Second, and more germane to the arguments we develop here, the assumption that conclusions in the natural domain about race are ambiguous are not in fact true at all. It is this second item of belief that we focus on in the rest of this paper.

## The ambiguity of biological race is ambiguous; the absence of biological criteria for race is unambiguous

Even after decades of examining the literature on the biological basis of race, many social scientists still seem to believe that ambiguity is inherent in the biological view of race. Hochman (2019) put it: “The biological race debate is at an impasse.” Such an impasse directly implies that the natural domain’s ontology of race is ambiguous; and, if this perception is accurate, it has profound implications for the philosophy of race. For Hochman (2019), for example, the notion that biological race is ambiguous forces one to approach the domain and mismatch problems in a very specific way, by arguing that this flight to deference (Hochman calls it “reference”) is unnecessary for social scientists who wish to proceed with developing their own ontology of race. We agree completely with this point, as we do with many of the arguments against a natural basis for race. And, as biologists familiar with the literature, we can easily see why Hochman (2019) and others have so eagerly latched on to this notion of an impasse. Nonetheless, we question whether that impasse actually exists. Of course, if one were to seek consensus on the subject of human race in the biological literature, one would court disappointment: papers and books regularly appear that both affirm and deny that human races exist in a biological sense. But the important point here is that science is not done by consensus. Rather, it is accomplished by a rigid objective process that even many practicing scientists do not fully understand. It is therefore imperative that we carefully examine the scientific and biological arguments for and against the reality of human races.

Here we immediately run into trouble because the concept of human races has been muddled since its inception. While the word “race” has been thrown around with abandon, it entirely lacks precise definition. This absence of precision has had horrendous historical consequences, as flimsy definitions and insidious uses of the concept have led to some of the most appalling episodes in the history of mankind. This ghastly record might reasonably lead one to question whether the race concept is of any utility whatever when applied to human beings; but people have nonetheless resolutely kept on using both the term “race” and the vague concept it denotes as if they were indeed somehow useful. One of the major reasons for this appears to lie in the belief that the term and concept are supported by science. It is this way of thinking that lies behind both the older “New Biology of Race,” and the more recent “racial realism” that has emerged from philosophical work in the last decade. Both approaches assume that race is a real biological phenomenon that explains human variation, and they can do so only because of the perceived impasse and the ambiguity it implies.

The endeavor of describing and explaining human variation is a tricky one that is littered with many misguided attempts at science. This has posed serious problems, because bad science runs the risk of adversely impacting non-scientific aspects of our existence that include our social, cultural and political beliefs. One common but misguided way of examining human variation involves the categorization of individuals into groups, based on what a particular researcher observes with respect to the variation observed. This kind of endeavor (we are reluctant to call it “science” unless certain qualifications are met) is full of pitfalls.

Valid science proceeds by a tried-and-true set of rules, perhaps the most precise of which came from the philosopher Popper (1963), whose writing attempted to summarize and validate his observations on the way scientists make progress. Popper’s approach has been labeled as “hypothetico-deductive”, since it posits that science proceeds by the generation of hypotheses and their subsequent testing. The relevance of the hypothetic-deductive approach to the practice of systematics has been discussed at length for several decades (Platnick, 1978; Ruse, 1979). While there are nuances with respect to a strict application of the approach to systematics (Rieppel, 2003; Helfenbein & DeSalle, 2005), Popper’s viewpoint is recognized as a good starting point for how science should generally proceed. In the hypothetico-deductive approach, if a hypothesis is rejected then another needs to be generated. If a hypothesis can’t be rejected, then a new and more restrictive hypothesis needs to be posed. Simply put, the hypothesis to be tested with respect to the existence of human races is the following:

H_0_ = there are N Human races corresponding to N geographic areas.

Geographic area is the simplest and most obvious way in which biologists lump their subjects of interest before actually gathering data about them. But other objectively defined criteria could also be used, such as a behavioral attribute, or some outward phenotype. For any test to be scientifically valid, it must be formulated and carried out objectively. If there is any ambiguity or subjectivity to the test, or to the conclusions made from it, then any “result” inferred from the test cannot be regarded as scientific. One might use the most up-to-date technical approaches to gather data for the test, and the most sophisticated analytical computational methods in vogue at the time; but if the overall criteria of the test, or of the analysis of the data derived from it, are in the least bit subjective or ambiguous, then any inference obtained is not scientific. A mere scientific slant to the test is not enough.

So how do we make sure that any testing of the hypothesis about human races given above is absolutely objective and unambiguous? Sober (2020) points out that, in taxonomy, the concept of monophyly provides an objective criterion for taxonomic tests: “A monophyletic group is comprised of an ancestor and all of its descendants, regardless of how different those descendants are from each other, and regardless of how different they are from their most recent common ancestor.” He goes on to note that “the idea that identifying monophyletic groups is the proper goal of taxonomy.” And while Sober leaves out diagnosis as the ultimate arbiter of taxonomic tests, we would add that the relationship of diagnosis and monophyly are entangled at several levels.

In making these points Sober was following the German systematist Willi Hennig (1999), who also showed that hierarchical relationships can only exist above the species level. What this means, is that any attempt to force hierarchy on the individuals within a single species is misguided. Reticulation destroys any hierarchy, and it is only with hierarchy that monophyly and diagnosis can occur. The suggestion that we can discover monophyly below the species level is thus illogical (Goldstein & DeSalle, 2000; Goldstein et al., 2000), as is any attempt to objectively define units within the species, such as race or subspecies.

Recently introduced genetic and genomic approaches have accentuated the debate over racial realism, but the problem has been around for a long time (Jackson & Depew, 2017), and physical anthropologists confronted the problems in the context of anatomical data for decades prior to the onslaught of genetic and genomic data. Fortunately, human genomic data are amply amenable to tests for monophyly and diagnosis. When such tests are done for monophyly, using tree building methods, the notion that race exists in human populations is soundly rejected (DeSalle et al., 2018). Some researchers have developed or adapted so-called quantitative techniques to “test” H_0_. These include STRUCTURE (here we are using STRUCTURE as a general term for a number of separate clustering approaches, most notably ADMIXTURE and fine STRUCTURE) and PCA. These data-reduction approaches have furnished tantalizing visual results that often provide unwarranted support of hierarchy; but they are not tests of H_0_. Edwards (2003) argued that these data reduction ways of analyzing genomic data can in fact extract taxonomic information. And while he did not say so explicitly, he implied that this is because data reduction techniques extract the underlying correlation structure of the data. This is an idea that Lewontin (1972) argued strenuously against, which is why Edwards’ suggestion has been called “Lewontin’s fallacy” (Edwards, 2003). Indeed, Hochman (2021a, b), for example, has argued that Lewontin did not create a fallacy with his locus-by-locus approach to understanding human variation, but that instead Edwards (2003) committed a fallacy of his own by implying that the underlying correlation structure can be used to test hypotheses about hierarchy. Suffice it to say that even the developers of the STRUCTURE and PCA methods recognize that these are methods for summarizing complex data sets, and not for testing hypotheses (Winther, 2014). They make no advance toward an unambiguous objective test of hierarchy (see DeSalle & Tattersall, 2018, 2023). In order to grasp this, one need only look at the results of a STRUCTURE analysis. If each of the hypothesized populations has 100% probable assignment of each member to that population, then hierarchy exists; and in that sense, STRUCTURE can be used as an unambiguous indicator of species existence. But below that distinctive level, any criterion that one places on any STRUCTURE analysis has to be subjective.

Our approach may sound very essentialist, and indeed it is. Hochman (2013) suggests that “The task for race naturalists, then, is to develop a biologically informed but non-essentialist concept of race.” This statement is revealing, because it recognizes that an essentialist test for anything below the species level is a pipedream. But we cannot envisage any criteria to define anything below the species level taxonomically or biologically, and hence any categories we may use below this level are necessarily nothing more than hypotheses. We suggest that there is no need to come up with a novel way to analyze human genomic data for hierarchal structure, other than diagnosis and monophyly. These latter tests, developed by systematists and taxonomists over the last 250 years (Mncube, 2015), are sufficient for us to implement a valid scientific test of any hypothesis about hierarchy; and in the end, we suggest that doing such tests objectively leads us ultimately to a logical argument against the existence of race as a biological concept. Our conclusion about race, from logic, is that there are no objective/unambiguous ways to test for the existence of biological race. Race is a term that was both invented and applied in the social domain, and it has no relevance to natural or biological reality.

## Relaxing things

Let’s relax our definition of a race to see if doing so allows for a solid unambiguous test of hypotheses. Race was first applied to biology as a part of the taxonomic hierarchy, in cases where the objectively defined taxonomic term “species” could not be applied to a group of individuals that a researcher felt, deep down, indeed represented a discrete lineage. That is not its definition, but rather the rationale for why the term was recruited for biological use. Another term invented by taxonomists to apply to groups of individuals about whose discreteness a researcher had a “gut” feeling, is “subspecies.” The reason that the subspecies category is ambiguous is that, unlike species, there are no tests of monophyly that can be used to objectively define it. Templeton (2013) has made the best attempt so far to define what a race is:

(i) A race is a geographically defined population within a species that has sharp boundaries that separate them from other populations.
(ii) A race is a distinct evolutionary lineage within a species characterized by a continuous line of descent.

Templeton (2013) emphasizes that “genetic differentiation alone is insufficient to define a subspecies or race under either of these definitions of race. Both definitions require that genetic differentiation exist across sharp boundaries and not as gradual changes, with the boundaries reflecting the historical splits. These sharp boundaries are typically geographic, but not always.” But note the somewhat subjective element in both ways in which Templeton defines a “race” above. In the first definition he uses the terms “sharp boundaries” and “separate.” What do these two terms mean? The word “sharp” seems to us non-objective. What might appear sharp to one researcher might be dull or inconsequential to another. “Separate” is another ambiguous term; what one researcher might think is “separate” might be a meaningless distinction to another. In the second definition the term “distinct” is ambiguous because it is imprecise. Contrast these uncertainties with the definition of a species in a taxonomic context, in which a species is a group of organisms possessing a diagnostic character or characters that differentiates it from other such groups. Or even with the biological species concept, that defines a species as a group of organisms reproductively isolated from other such groups. Although it has operational issues, the biological definition is incredibly objective; if two groups of organisms don’t completely exchange genetic material with each other, then they are distinct from each other.

In contrast, the racial epithet and its use in taxonomy has been very difficult to pin down. Even the subspecies is a difficult entity to pin down objectively in a taxonomic context. The term subspecies was developed to refer to potential species that required attention from the taxonomist, so that the term “subspecies” refers more to a hypothesis than to a test of a hypothesis. In the context of taxonomy, the term “race” was coined to refer to potential groups in much the same way, but with even less definition than subspecies.

We are not arguing with Templeton’s definitions, which are as good as it gets with respect to races. But we do want to point out that these definitions – and indeed any definition that we can conjure up for race, and for subspecies for that matter – cannot provide an unambiguous basis for a scientific test. Races and subspecies can be used as hypotheses for the existence of a real group, but they are not real entities in nature. Quite simply, the rules of taxonomy were not developed to accommodate any group below the species level. Some might argue that this is a semantic issue, but we suggest that it is a serious theoretical point that renders objective discovery of races or subspecies unattainable. A final note on Templeton’s definitions is that, even though the criteria for hierarchy are relaxed and somewhat ambiguous, Templeton was able to reject all attempts at discovering human races using his softer criteria. While this is not a test of a scientific hypothesis, it is obvious that if relaxed criteria for discovering races are rejected, then any stronger unambiguous definition we can derive will also be rejected, simply with more rigor.

## Race, racial, racialized, racialist, racist, racial realism and reification

We hope it has already become clear that, at least in the natural domain, race is an ontologically faulty term and concept. But is racial biology necessarily a bad thing? To this we have to answer in the negative, for we cannot scientifically test H_0_ without taking a racial approach to the question. And it is important to note that, while racialized, this approach is not racist. It would, of course, be racist if H_0_ were to be tested and rejected, and nonetheless made part of the philosophy of race. But racial approaches to social sciences studies, and to studies of the environmental effects on traits in the natural domain, are clearly important, as is the unfortunate fact that race is currently entrenched in our human perceptions. This has led many researchers to claim that, if we ignore race, then we are ignoring racial realism. But then the question arises: racial realism in which domain? The danger of a faulty approach to race realism inevitably results in racial reification (Duster, 2005; Gannett, 2004; Hochman, 2021a, b; Dimick, 2023; Glasgow, 2015), or the process of promulgating and utilizing the existence of races in the face of evidence to the contrary. Prominent examples of racial reification can be found in forensics testing and direct to consumer genomics (Skinner, 2020; M’charek et al., 2020; Zarrugh & Romero, 2023).

In 2015, Spencer (2015) examined “racial realism” from a philosophical standpoint, and for many of the same reasons that caused Hochman to characterize biological studies of race as being at an impasse. Spencer was worried that broad rejection of racial reality in the natural domain would be damaging to an ontology of race (Spencer, 2012, 2014); and to clarify his argument he pointed to several problems that arise when racial realism is rejected unwarrantedly. The first two of Spencer’s four perceived problems are semantic, and the latter two are metaphysical (DeSalle & Tattersall, 2023). In the following remarks we paraphrase and comment on each of them:

- The “discreteness objection”: According to Spencer, anti-racial realists conclude that no level of human population structure can show racial groupings, because the hypothesis that a race exists means that we need to test for discreteness. We point out that, yes indeed, discreteness (monophyly or diagnosis) is the only objective criterion for this test; but the objection itself is semantically faulty because discreteness cannot be a consideration in a species in which many people identify to more than one race. Precisely because many people identify to more than one race, diagnosis and monophyly are not discoverable in the human species.
- The “visibility objection”: This objection maintains that no manner of structuring or stratifying human populations will show racial groupings, because such groups should have visible distinctive traits. In the philosopher Joshua Glasgow’s (2003) words, it is “conceptually non-negotiable, that each race by and large has a distinctive set of visible traits.” In other words, visible traits and potential racial groups do not overlap. This argument is semantically faulty because members of the same race do not necessarily have traits that define them.
- The “very important objection”: This objection suggests that no level of human population structure contains real racial groups because, to be biologically “real,” such groups need to be important enough to be classified biologically. As we have seen (see our discussion of Templeton’s definitions of race), the taxonomic term “race” is sufficiently subjective for such groupings to be rejected. Lewontin (1972) first used this argument to reject biological races; Edwards (2003) rejected it as “Lewontin’s fallacy;” and Hochman (2021) reinstated it by pointing out that Edwards, rather than Lewontin, had proposed a fallacy.
- The “objectively real objection” states that no level of human population structure is biologically real, because those levels are not themselves objectively real. Why? Because they would not exist if it were not for human interest, belief, or some other human psychological state. Spencer rejects this objection as follows: “in order to accurately capture the real entities of human biology, we need to embrace biologically real entities whose existence is dependent on human mental states, such as human beliefs and human interests.” In other words, we need the products of human mental states to formulate hypotheses.

Spencer’s refutation of these objections by the opponents of “racial realism” are interesting, but we feel that there is conflict here with the scientific process of hypothesis testing that we discuss next. We agree with Spencer that the rejection of racial realism cannot be based on faulty logic, for misleading conclusions result when there is a misunderstanding of the practice and goals of taxonomy. And Spencer’s arguments assume that the data actually show discrete differentiation, which is not at all true.

We also feel compelled to point out a problematic aspect of publications such as Charles Murray’s (2020) *Human diversity: The biology of gender*,* race*,* and class*, and his 2021 book *Facing reality: Two truths about race in America* (Murray, 2021). Note that the word “reality” in Murray’s title reflects an absolute claim for the contents of his book – a deference to racial realism. Almost a decade earlier, Wade (2015) had made the same claim, but in a different way that was particularly patronizing to scientists. Wade suggested that if something is scientifically sound, then we are wrong to ignore it. We could not agree more with Wade on this principle, but sadly his exposition of the genomic data in support of the existence of races was seriously flawed in many of the ways we have discussed above. Many others who have jumped into the fray of the philosophy of race have also done so as self-identified “race realists.” Their argument goes like this: “You scientists know that race is real from the data, and your tendency to ignore it is unscientific.” While it is true that ignoring accurate scientific results is to willfully abandon the scientific process, those results need to be accurate in the first place, which is where all the race realism arguments fall flat. The conclusion that races are biologically real is simply false, and to continue to test this unequivocally false and already discredited hypothesis is problematic.

The principal mistake that race realists make, then, is to claim that they are doing or invoking science. As described earlier, science proceeds by hypothesis testing. And while race realists will argue that they have tested a hypothesis (i.e., “H_0_ = Races exist”) this to our knowledge is always not the case. We have detailed the mistakes made by race realists in this respect (DeSalle & Tattersall, 2018; DeSalle & Tattersall, 2023) and list them again in Table 1.

**Table 1:** Seven mistakes race realists make when claiming a new biology of race

Mistake	Comment
Misunderstanding hypothesis testing.	This is the most common mistake made by race realists.
[straighten up] Misunderstanding the biological meaning of taxonomic terms	Unlike species, in which the definition is objective and operational, the definition of race as a taxonomic epithet is not.
Misunderstanding the meaning of cluster analysis.	While these analytical methods are widely used by researchers who study human variation, they do not test hypotheses about the biological existence of human races.
Lack of consideration of bias when using data-reduction techniques; cherry-picking data sets.	Sequence ascertainment methods need to be more objective
Being fooled by the “underlying correlation structure” of human traits and genetics.	The claim that a diagnostic exists from the underlying correlation structure of a group of people is unscientific.
Conflating racially based genetic differences with explanation of ancestry	Ancestry and race are NOT the same thing
Conflating geographically skewed allele frequency differences with adaptation.	This misunderstanding is takes a flawed positive inference about race and uses it in a racist argument

## The new biology of race is the same as the old

We find the philosophy of race approach to an ontology of race to be a potentially productive way to view race in contemporary society. As we have pointed out, in order to proceed to a precise ontology the natural and social domains of knowledge need to be individually recognized, and properly treated. Proponents of the philosophy of race have dealt with this domain problem by breaking it down into two more precise problems: deference and mismatch. We conclude here, however, that the only way in which social science needs to defer to natural science in developing an ontology is to recognize that race is neither a natural nor a biological concept. And while several authors have argued that the natural domain is at an impasse, we show that under any criteria imaginable the natural domain rejects H_0_. What is more, in doing this we inevitably discover that there simply is no objective way to test H_0_ at the level of race.

To put all this another way, as Andreasen (2020) has stated, a good natural definition of race has four components to it:

1. Race is a subdivision of *Homo sapiens*.
2. Observable physical features are often used to sort people into races.
3. The members of race are often linked by common ancestry.
4. The members of a race often originate from a distinctive geographic origin.

Andreasson’s components are very similar to (but not as precise as) those formulated by Templeton (2013). And, as with Templeton’s definition, they suffer from subjective criteria embedded within them. Again, if there is no objective test for race then any attempt at defining a biological race becomes, by definition, entirely arbitrary. Which in turn eliminates the biological domain from ontological consideration in formulating a philosophy of race. If such a test could be devised, it would indeed connect the two domains (as race philosophy has done by allowing biology to inform the social kind of race – and, in some ways, vice versa). However, we cannot see how a non-arbitrary definition of race could ever exist, due to the following biological realities relevant to sexually breeding organisms:

1. Breeding across our species destroys the hierarchy necessary for an objective set of criteria for race and even subspecies.
2. Reticulating mating systems like our own might have certain characteristics that come close to being hierarchically distributed; but since the reticulation is real, the criteria for definition will never be attained.
3. No biological criterion is sufficiently objective to be used as a non-arbitrary tool for identifying someone unambiguously to a race. Despite claims in forensic science that such tools exist, they all break down and will eventually become useless with more and more reticulation.
4. While there are some algorithms that purport to decide on a species boundary between two entities that have not passed the diagnosis test, these approaches are always arbitrary in their assumptions. They require model parameters and models that approach reality but are ultimately arbitrary. If one could establish non-arbitrary models (this is impossible by definition) and model parameters (again, by definition not objective: their whole point is to explore a data space subjectively), then we suppose we could have a non-arbitrary definition of race. But because arbitrariness is built into models and parameterization, this will not occur.
5. All tools developed so far for characterizing population level “hierarchy,” while useful in understanding trends of divergence and recontact in the populations, give arbitrary results. This includes distance-based trees, STRUCTURE, STRUCTURE-like algorithms and PCA. Changes in criteria can be introduced into all these approaches, making them less arbitrary, but in the end the nature of these kinds of analyses is such that they can never be fully objective.

So, will there ever be a non-arbitrary (objective) scientific test for race? Can we develop a set of criteria for race, or even a single criterion that is as objective as the test for species? Or, for that matter, can we propose criteria for the biological entity we call subspecies? We suggest that the answer to these questions is an empirical and emphatic “No.” Even pressing the limits about the way we observe organisms in biology, we are at a loss to specify a single biological property of organisms that might offer an objective way to test for the existence of race. At the same time, all objective criteria or techniques we can apply to human genomic data to extract hierarchical structure (tree-building or cluster analysis) invariably reject the null hypothesis. Hierarchy cannot therefore be considered an element of an ontology of race. Human variation exists; biological races don’t. One might be able to test for the existence of isolation in the past, or to predict that two entities will become isolated in the future; but any philosophy of race ontology is necessarily about the present. We conclude, then, that we can and must reject the null hypothesis that human races exist. This rejection means that a philosophy of race needs to recognize the non-reality of biological race as part of the ontology. This rejection also means that deference to the natural domain in developing an ontology for a philosophy of race should include the lack of evidence for biological races. There is no need for the social sciences to defer in any other context to the natural sciences on the subject of race. Races are artefacts of human perception, and thus exist purely in the social domain. Trying to enlist a racial realist component from science into the philosophy of race – one way or the other–is illogical, unhelpful and at worst profoundly misleading and destructive.
